# Effectiveness of the credit-line approach for support of CD4 equipment functionality in northern Uganda

**DOI:** 10.4102/ajlm.v4i1.234

**Published:** 2015-11-30

**Authors:** Michael L. Kasusse, Nazarius M. Tumwesigye, Steven Aisu, Joseph K.B. Matovu, Rhoda Wanyenze

**Affiliations:** 1 Makerere University School of Public Health, Kampala, Uganda; 2 MakSPH-CDC Fellowship Program, Makerere University School of Public Health, Kampala, Uganda; 3 Central Public Health Laboratories, Ministry of Health Uganda, Kampala, Uganda

## Abstract

**Background:**

Improving laboratory service delivery requires a functioning logistics and supply system. Uganda’s Ministry of Health uses the credit-line approach to provide laboratory supplies including commodities for CD4 test equipment.

**Objectives:**

We examined the effectiveness of the credit-line approach in improving laboratory service delivery by using the functionality of CD4 test equipment as a proxy indicator.

**Method:**

A cross-sectional survey was conducted at 7 level-three health centres (HC IIIs), 18 level-four health centres (HC IVs), and 10 hospitals in 15 districts of mid-northern Uganda, including the Lango (17 facilities) and Acholi sub-regions (18 facilities), between July 2013 and August 2013. Functionality, was determined through self- and interviewer-administered questionnaires. The chi-squared test was used to assess differences in functionality by sub-region, facility type, and equipment type.

**Results:**

A total of 38 CD4 test analysers were assessed. Of these, 26 (68%) were functional. In hospitals, 85% of CD4 analysers were functional, in HC IVs, 67% were functional and in HC IIIs, 43% were functional. The differences did not reach statistical significance. In the Lango sub-region, 72% of analysers were functional; in the Acholi sub-region, 65% were functional. Non-functionality was mainly due to lack of reagents and cartridges, as well as low staffing levels of laboratory technicians with the skills necessary to operate the equipment.

**Conclusion:**

The credit-line approach supported the functionality of CD4 equipment in the surveyed facilities. However, there is a need to address issues of staffing and availability of reagents to enhance the functionality of CD4 equipment and improve patient care, especially at HC IIIs.

## Introduction

Until 2003, the Ministry of Health (MoH) of Uganda used a ‘push’ system to guarantee supply of medical commodities, especially in areas affected by natural disasters or areas such as northern Uganda, which was affected by a 20-year-long civil war. In the push system, the authorised supplier determines the types and quantities of supplies to be issued to health facilities.^1^ Inconsistencies remained between the needs of the user areas and the medical items supplied. In many health facilities, there were frequent stock outs of supplies (including reagents for CD4 equipment) and large quantities of expired items (including cartridges for CD4 equipment).^2^ As a consequence, this led to non-functioning equipment and patients missing CD4 monitoring. The MoH introduced a ‘pull’ system in 2003 to overcome the challenges of frequent stock outs and expired items. This system required health facilities to determine the types and quantities of medical items that they needed.^3,4^ An assessment of the performance of the pull system to improve the availability of equipment and reduce expiration of medical supplies showed that the pull system improved the availability of medical supplies, but did not address challenges such as inadequate training of staff, lack of transport and inadequate funding.^5^

In order to address the challenges of funding and transport, the MoH introduced the ‘credit-line’ approach in 2009 at the government-owned National Medical Stores supported by development partners, including the United States Centers for Disease Control and Prevention, Uganda; the Clinton Foundation Health Access Initiative, Uganda; UNITAID; and Global Fund, amongst others. Of the funding available, 80% was allocated to public health facilities and 20% to private not-for-profit health facilities.^6^

This approach requires that, during every cycle of two or four months, each health facility is allocated a financial vote, known as a credit line, from which to draw an equivalent of equipment and supplies. The MoH’s Central Public Health Laboratories (CPHL), the laboratory activities unit, identifies a list of supplies, recommends specifications, quantifies and makes forecasts that cater to the credit line for each facility. Laboratory commodities and supplies, including CD4 equipment commodities, are procured and distributed or transported to the districts by the National Medical Stores for public facilities and by a private, not-for-profit store, the Joint Medical Store, for public, not-for-profit facilities. Each health facility is tasked with placing orders, picking up the supplies from the districts, maintaining consumption data and sending the data to CPHL for use in making other forecasts.^6^ However, the role of this approach in supporting the functionality of facilities’ CD4 equipment has not been evaluated in northern Uganda.

Laboratory monitoring of HIV patients determines eligibility for anti-retroviral treatment (ART), which slows down the progression of HIV to AIDS, in addition to monitoring the efficacy of ART after initiation.^7^ It has been shown that, compared with viral load, CD4 count is a better predictor of clinical progression of HIV to AIDS and is a better guide for initiation of ART.^8,9^ In low-income countries, laboratory monitoring of patients on ART remains controversial because of ongoing resource limitations.^10^ In addition, unreliable or inaccurate testing leads to unnecessary costs in areas that already experience shortages. This, in turn, leads to the perception that laboratory testing is unhelpful or that it could compromise patient care.^11^

In developing countries, CD4 testing is common in urban areas, where patients can undertake multiple visits to clinics. The expansion of ART programmes into rural areas created a need for point-of-care (POC) CD4 testing in order to overcome logistical barriers in the timely dissemination of test results and initiation to care programmes.^12^ Rapid, reliable and affordable POC CD4 tests are not yet widely available.^13,14^ Some of the POC CD4 test technologies on the market or in development include: PointCare^®^ NOW; CyFlow^®^ miniPOC; PIMA™ CD4; Coulter CD4 Count Kit; Dynal^®^ T4 Quant Kit; Daktari CD4; MBio^®^ CD4 System; Visitect^®^ CD4; and Zyomyx^®^. Some of these are being used in the northern region of Uganda.^15^

POC CD4 testing is an efficient intervention to reduce pre-treatment loss to follow up, because it enables clinics to stage patients rapidly on site, so that more patients are determined to be eligible and initiated on ART.^16^ The aim of diagnostic POC testing is to minimise the wait time to obtain a test result, allowing clinicians and patients to make a quick clinical decision. In resource-limited settings, the benefits of POC testing outweigh its costs by focusing on the relevant clinical outcomes.^17^

There is also a need for laboratory follow-up of HIV patients in resource-limited settings, if appropriate CD4 test equipment is used. The Auto 40 system, which uses thermoresistant reagents, is one example of a CD analyser that is appropriate for such settings.^18^ The MoH’s credit-line approach is intended to ensure adequate supply of such equipment and associated reagents for clinical monitoring of patients with HIV.

We evaluated the effectiveness of the credit-line approach for improving laboratory service delivery to healthcare facilities in northern Uganda. Facilities were surveyed to determine the functionality of their CD4 equipment as a proxy indicator of the effectiveness of the approach, including reasons for non-functionality. Various factors, such as facility type and staffing levels, were examined to suggest ways of improving laboratory service delivery in the country.

## Methods

**FIGURE 1:**
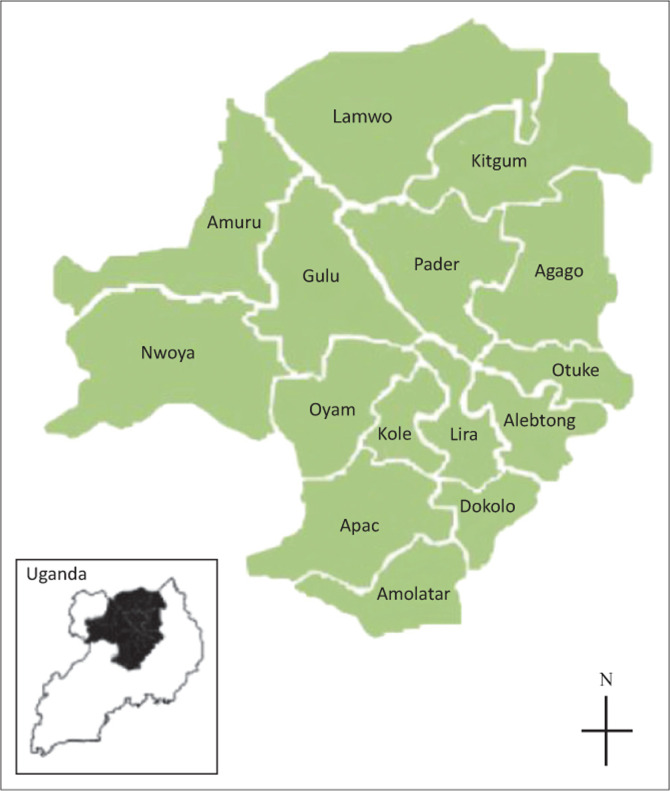
Area of survey: Acholi and Lango sub-regions of Uganda.

As part of the National Laboratory Supplies Quantification and Verification exercise, between 01 July 2013 and 02 August 2013, the MoH’s CPHL conducted a cross-sectional survey at health facilities in the Lango and Acholi sub-regions of mid-northern Uganda. The Lango sub-region includes eight districts and the Acholi sub-region, seven districts. Facilities in the study area included the following types ordered by complexity of service delivery: private for-profit; private not-for-profit and government or public clinics, including level-two health centres, which are closest to communities and offer basic health services; level-three health centres (HC IIIs); level-four health centres (HC IVs) which offer more complex services similar to hospitals; general hospitals; and regional referral hospitals. At least one government laboratory facility was included for each of the 15 districts of mid-northern Uganda (Figure 1). Laboratories in an HC IV facility were selected preferentially, because the MoH plans to transform HC IVs into laboratory hubs.

Depending on the availability of the laboratory managers (or ‘in charges’), semi-structured questionnaires were either self-administered by the laboratory managers or interviewer-administered to the available laboratory staff by the research team. The research team, which was composed of three CPHL technical staff, including a team leader and a driver, travelled to each facility to distribute and collect the questionnaires. The questionnaires gathered the following information: the type and location of CD4 equipment and functionality; reasons for non-functionality; and staffing levels for each cadre, including laboratory technologists, laboratory technicians, laboratory assistants and microscopists, by facility.

Completed questionnaires were first reviewed in the field by the research team to ensure completeness and accuracy. The questionnaires had an optional telephone contact field and collectors contacted the respondents to validate information that was not clear. The Statistical Package for the Social Sciences statistical software (version 17.0; SPSS Inc., Chicago, IL 2008) was used for data entry and analysis. The chi-squared method was used to evaluate whether there was a statistically-significant difference between the functionality of CD4 equipment by sub-region, facility level or equipment type. The outcome variable for determining the effectiveness of the credit-line approach was whether the CD4 equipment at each facility was functional. Functional was defined as CD4 equipment that was capable of carrying out CD4 tests. The credit-line approach was considered to be effective for facilities with functional CD4 test equipment. We also evaluated whether facility level, distribution and type of CD4 equipment or the staffing levels by facility affected the presence of functional CD4 equipment. *P*-values below the conventionally accepted significance level of 0.05 (or 5%) were considered to be statistically significant.

## Results

A total of 16 self-administered questionnaires were completed by the managers of laboratory facilities. Laboratory managers were absent at 19 laboratory facilities, at which the research team conducted interviewer-administered questionnaires with available staff. Thus, of the 68 public facilities in the Lango and Acholi sub-regions, a total of 35 facilities were assessed (Table 1), including 7 HC IIIs, 18 HC IVs and 10 hospitals in the Lango (17 facilities) and Acholi (18 facilities) sub-regions. These 35 facilities included 38 CD4 analysers – 18 in the Lango sub-region and 20 in the Acholi sub-region. PIMA was a predominant type of CD4 equipment and the majority of CD4 analysers were in HC IV facilities (18 of 38; 48%).

Overall, 26 of the 38 (68%) CD4 analysers were functional (Table 2). Although the Lango sub-region had fewer CD4 analysers than the Acholi region, a higher percentage were functional (72% in Lango vs. 65% in Acholi). Similarly, although there were more CD4 analysers at HC IV facilities, hospitals had a higher percentage of functional CD4 equipment (67% in HC IV facilities vs. 85% in hospitals); HC IIIs had the lowest percentage of functional CD4 analysers (43%). There were no significant differences in the functionality of CD4 equipment by sub-region, facility level or equipment type.

Non-functionality of CD4 equipment was mainly because of the lack of reagents or cartridges (Table 3). Other reasons given included expired cartridges, unpredictable power sources and lack of controls. Low staffing levels were reported amongst the various laboratory cadres with the skills necessary to operate the equipment (Table 4). The lowest levels were observed amongst laboratory technologists at hospitals (6 of 31 positions filled; 19%) and laboratory assistants at both HC III (4 of 12 positions filled; 33%) and HC IV facilities (19 of 70 positions filled; 27%).

**TABLE 1:** Distribution of CD4 analysers at healthcare facilities surveyed in northern Uganda, July–August 2013.

Characteristics	HC	III	HC IV	Hospitals	Total
Total number of facilities	7	18	10	35
**Sub-region**				
Acholi	4	7	9	20
Lango	3	11	4	18
Total number of analysers	7	18	13	38
**Districts of Acholi**				
Agago	1	-	1	2
Amuru	-	1	-	1
Gulu	-	2	3	5
Kitgum	-	1	3	4
Lamwo	-	2	-	2
Nwoya	1	-	1	2
Pader	2	1	1	4
Total number of analysers	4	7	9	20
**Districts of Lango**				
Alebtong	-	1	-	1
Amolatar	1	2	1	4
Apac	-	1	1	2
Dokolo	1	1	-	2
Kole	-	1	-	1
Lira	-	3	-	3
Otuke	-	1	-	1
Oyam	1	1	2	4
Total number of analysers	3	11	4	18
**Equipment type**				
PIMA	7	18	3	28
BD Facs Count	-	-	6	6
Guava Technology	-	-	1	1
Partec Cyflow	-	-	1	1
Point Care	-	-	2	2
Total number of analysers	7	18	13	38

HC III, level-three health centre; HC IV, level-four health centre.

## Discussion

This assessment of the credit-line approach for providing laboratory supplies (including commodities for the functionality of CD4 equipment) to health facilities found that 68% of CD4 equipment was functional, despite the low staffing levels of laboratory cadres by facility level. Staffing issues related to provision of commodities and supplies still play a role in the effectiveness of the credit-line approach, especially in areas with a history of war and natural disasters.^3,4,5^ Despite persistent resource constraints,^10^ POC CD4 testing is being implemented successfully in northern Uganda to overcome logistical barriers to the timely dissemination of test results and initiation to care programmes.^12^

**TABLE 2:** Functionality of CD4 equipment by region, facility level and equipment type.

Variable	Functionality				Chi-square statistics	
	Yes		No			
	Number	Percentage	Number	Percentage	χ^2^	*p*-value
All	26	68	12	32	-	-
**Sub-region**					χ^2^ = 0.22	*p* = 0.63
Acholi	13	65	7	35	-	-
Lango	13	72	5	28	-	-
**Facility level**					χ^2^ exact = 3.7	*p* = 0.16
HC III	3	43	4	57	-	-
HC IV	12	67	6	33	-	-
Hospital	11	85	2	15	-	-
**Equipment type**					χ^2^ exact = 0.84	*p* = 0.36
PIMA	18	64	10	36	-	-
Others†	8	80	2	20	-	-

HC III, level-three health centre; HC IV, level-four health centre.

† BD Facs Count; Guava Technology; Partec Cyflow; Point Care.

### Limitations

The credit-line approach in Uganda is being supported by developing partners to provide commodities and supplies to public and private not-for-profit health laboratories for the functioning of CD4 equipment.^6^ This approach excludes specialised testing at high-level reference laboratories, which play a very important role in outbreaks and disease surveillance in the country. When interpreting our results, readers should also consider the lack of baseline data to use as a comparison between the push and pull systems with the credit-line approach; the fact that the selection of laboratories included in the analysis was not random; and that the intended respondents (laboratory managers) were not available for more than half of the surveyed laboratories where interviewer-administered questionnaires were instead conducted with laboratory staff members.

### Recommendations

There is a need to address issues of staffing and availability of reagents to enhance the functionality of CD4 equipment and improve patient care, especially at HC IIIs. Staffing issues related to the functionality of CD4 equipment used in the clinical monitoring of HIV patients, such as provision of commodities and supplies, should be regarded as highly important in the effectiveness of the credit-line approach. Such issues may include recruitment, as well as retention and training. There is also a need to include specialised facilities in the credit-line approach systems for meaningful laboratory services in Uganda.

### Conclusion

The credit-line approach supports the functionality of CD4 equipment used in the clinical monitoring of HIV patients in areas with a history of war and natural disasters, such as northern Uganda. Staffing issues related to provision of commodities and supplies play a role in the effectiveness of the credit-line approach.

**TABLE 3:** Reasons for non-functionality of CD4 equipment.

Reason	HC III	HC IV	Hospitals	Total
No cartridges	2	2	-	4
No reagents	-	3	2	5
Expired cartridges	-	1	-	1
Power source	1	-	-	1
No controls	1	-	-	1

HC III, level-three health centre; HC IV, level-four health centre.

**TABLE 4:** Staffing levels of facilities by cadre.

Cadre	HC III			HC IV			Hospitals		
	No. of positions filled	No. of positions available	Percentage of positions filled	No of positions filled	No. of positions available	Percentage of positions filled	No. of positions filled	No. of positions available	Percentage of positions filled
Lab technologists	1	1	100	1	1	100	6	31	19
Lab technicians	7	8	88	17	34	50	20	30	67
Lab assistants	4	12	33	19	70	27	30	46	65
Microscopists	1	1	100	2	2	100	7	7	100
Staffing totals	13	22	59	39	107	36	63	114	55

HC III, level-three health centre; HC IV, level-four health centre.
